# Macro optical projection tomography for large scale 3D imaging of plant structures and gene activity

**DOI:** 10.1093/jxb/erw452

**Published:** 2016-12-26

**Authors:** Karen J. I. Lee, Grant M. Calder, Christopher R. Hindle, Jacob L. Newman, Simon N. Robinson, Jerome J. H. Y. Avondo, Enrico S. Coen

**Affiliations:** 1 John Innes Centre, Norwich Research Park, Colney Lane, Norwich, NR4 7UH, UK

**Keywords:** 3D, development, gene activity, imaging, macroscopic, macro optical projection tomography, M-OPT, plant-insect interactions, tomography, whole plant.

## Abstract

Optical projection tomography (OPT) is a well-established method for visualising gene activity in plants and animals. However, a limitation of conventional OPT is that the specimen upper size limit precludes its application to larger structures. To address this problem we constructed a macro version called Macro OPT (M-OPT). We apply M-OPT to 3D live imaging of gene activity in growing whole plants and to visualise structural morphology in large optically cleared plant and insect specimens up to 60 mm tall and 45 mm deep. We also show how M-OPT can be used to image gene expression domains in 3D within fixed tissue and to visualise gene activity in 3D in clones of growing young whole Arabidopsis plants. A further application of M-OPT is to visualise plant-insect interactions. Thus M-OPT provides an effective 3D imaging platform that allows the study of gene activity, internal plant structures and plant-insect interactions at a macroscopic scale.

## Highlight

Macro OPT captures 3D morphologies of plants up to 60 mm by 45 mm, enabling visualisation of plant-insect interactions and gene activity.

## Introduction

To understand how genes regulate changes in the shape of growing plants it is essential to be able to visualise them in three dimensions. Imaging deep into larger plant specimens reveals the 3D spatial relationship between gene activity and plant growth. Advances in confocal and light sheet microscopy have enabled the visualisation of gene expression at cellular and subcellular levels in living plant tissues (Verveer *et al.*, 2007; Truernit *et al.*, 2008; Wuyts *et al.*, 2010; Kurihara *et al.*, 2015; Ovecka *et al.*, 2015; Bassel and Smith, 2016). However these optical techniques remain limited for 3D visualisations at depth. In comparison, optical projection tomography (OPT) is a 3D imaging method that allows the visualisation of gene expression patterns to a greater depth but at a lower resolution (Sharpe *et al.*, 2002; Lee *et al.*, 2006). OPT has been successfully used to visualise gene activity during animal and plant organ development, and it is most successfully applied with optically cleared fixed specimens. OPT can also be used with small semi-transparent live animal organs (Boot *et al.*, 2008; Colas and Sharpe, 2009; Arranz *et al.*, 2014) and plants. However most commonly used OPT systems, such as the Bioptonics Scanner 3001 and Medical Research Council (MRC) Prototype OPT scanner, are limited to a sample size of between approximately 0.5 mm and 15 mm, with a spatial resolution of 1.6 to 40 µm/voxel. They are therefore unable to image whole plants or large flowers.

In recent years a number of open source custom OPT systems have been built (Gualda *et al.*, 2013; Wong *et al.*, 2013) and OPT based scanners and software have been developed to increase spatial resolution and visualise smaller structures (Fauver *et al.*, 2005; Walls *et al.*, 2005; Miao *et al.*, 2009; Rieckher *et al.*, 2011; Fei *et al.*, 2012; Chen *et al.*, 2013; Pardo-Martin *et al.*, 2013; Mayer *et al.*, 2014). However, apart from helical OPT (Arranz *et al.*, 2013), which allows 3D image acquisition of long specimens, development of these systems has not applied OPT imaging to large specimens at greater depths.

Other 3D imaging systems which have overcome the depth limitation of OPT exist but they have other restrictions. For example, high resolution X-ray computed tomography (HRCT) enables living plants to be imaged (Stuppy *et al.*, 2003; Kaminuma *et al.*, 2008; Dhondt *et al.*, 2010; van der Niet *et al.*, 2010; Dorca-Fornell *et al.*, 2013; Pajor *et al.*, 2013; Verboven *et al.*, 2015; Wang *et al*., 2015). Multi-resolution HRCT scanners are capable of visualising plant samples ranging from 37 cm in diameter with a spatial resolution of 100 µm/voxel to specimens 200 µm in diameter at 400 nm/voxel (Dhondt *et al.*, 2010). However, the low X-ray absorption of plant tissues makes some internal structures difficult to visualise by HRCT (Staedler *et al.*, 2013). Additionally, gene expression cannot be visualised and X-rays can adversely affect plant growth (Dhondt *et al.*, 2010). Magnetic resonanace imaging (MRI) can also be applied to non-invasively visualise internal plant structures in 3D and can identify metabolites and track the movement of liquids in large growing plant tissues (Melkus *et al.*, 2009; Windt *et al.*, 2009; Mooney *et al.*, 2011; Borisjuk *et al.*, 2012; Flavel *et al.*, 2012; Kenouche *et al.*, 2014; Metzner *et al.*, 2014; Metzner *et al.*, 2015). However MRI, like HRCT, cannot be used to visualise gene expression and is generally applied to explore liquid transport and water content in roots and stems rather than visualising detailed internal plant structures. Thus neither HRCT nor MRI can be used to capture gene expression patterns in plants.

Here we describe the first custom built Macro OPT (M-OPT) scanner which enables the visualisation of internal structures in optically cleared specimens up to 45 mm deep and 60 mm tall. M-OPT is applied to observe gene expression in both fixed and growing flowering Arabidopsis plants with roots and to explore spatial relationships in developing plant organs and between plants interacting with insects.

By expanding the sample size, larger scale OPT could enable gene expression to be visualised in 3D to the depth of a small whole Arabidopsis plant. Gene activity in leaf surfaces and roots in the same growing plant could therefore be tracked. Internal structures in large plant organs such as flowers could be resolved allowing floral developmental series to be completed and interactions between insects and plants to be observed in 3D. These aspects are not possible with currently available imaging technologies.

## Materials and Methods

### Specimen preparation and mounting

**Fig. 1.**
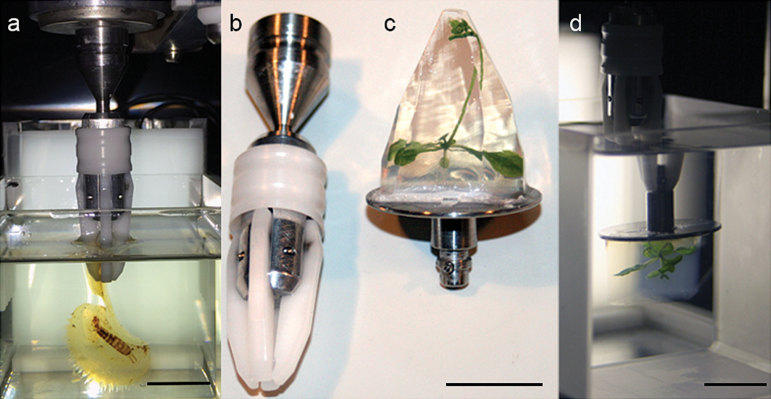
Sample grabber, adaptors and cuvette. (a) Cuvette with cleared Venus fly trap (*D. muscipula*) and earwig (*F. auricularia*) directly mounted with a grabber in a cuvette filled with BABB. (b) Grabber. (c) Adaptor mount with agarose embedded Arabidopsis plant for live OPT. (d) Live Arabidopsis specimen in a cuvette filled with water. Scale bars, 15 mm.

**Fig. 2.**
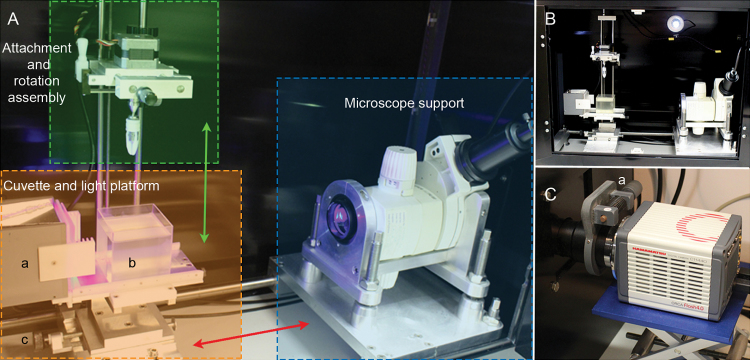
The M-OPT system. (A) Sample attachment and rotation assembly highlighted in green moves vertically (green arrow). The cuvette and LED light platform (sepia highlight) consists of (a) LED lights for transmission illumination, (b) 70 mm quartz cuvette, (c) focus control, which moves horizontally to focus the specimen (red arrow). Microscope with fluorescence illumination is highlighted in blue. (B) M-OPT components are encased in a black box to exclude light. (C) Camera mount is located outside the light-excluding box and connects the camera and camera rotation device (labelled ‘a’) to the microscope.

Conventionally, specimens for OPT are embedded in agarose before clearing and mounting for OPT image collection. However, we found these agarose blocks become too heavy and unwieldy when mounting large specimens. To address this problem, we developed a grabber with attachments, a flexible method allowing mounting of both large samples with stems and smaller tissues embedded in agarose in the M-OPT scanner (Fig. 1). The grabber, used in conjunction with a large cuvette, high resolution camera and adapted microscope (Fig. 2), enables collection of images with a wide field of view. This facilitates M-OPT visualisation of plant organs up to 60 mm tall and 45 mm deep (Fig. 4).

Specimens up to 35 mm tall can be embedded in 1% low melting point agarose for support and stability. The agarose block is superglued to an adaptor mount and excess agarose trimmed away (Fig. 1c). The mounted sample is dehydrated in methanol for up to a week and optically cleared in Murray’s clear (2-parts benzyl benzoate/ 1-part benzyl alcohol (BABB)) until the tissue is semi-transparent. The specimen is attached to the M-OPT scanner via a grabber mount (Fig. 1b) and rotated through 360^o^ in a 70 mm custom made quartz cuvette (Multi-Lab, Ltd, Newcastle upon Tyne, UK) containing BABB to maintain equal refractive indices between the sample and scanning medium. For live M-OPT imaging, whole Arabidopsis plants up to 20 mm by 20 mm by 35 mm are embedded in agarose and M-OPT scanned in water without optical clearing (Fig. 1c, d)

Larger samples not embedded in agarose are attached to the scanner via a grabber, either directly or with adapters (Fig. 1a). Specimens are dehydrated in methanol overnight and cleared in BABB. Clearing takes a variable amount of time, from 24 hours to several weeks, depending on the depth of the specimen and opaque compounds present. For combined plant and insect tissue clearing we found insect exoskeletons could take up to 12 months to become transparent (Fig. 6h-j). To solve this problem, Visikol (Phytosys, Somerville, USA) proved to be an effective clearing agent (Villani *et al.*, 2013). A *Dionaea* trap containing an earwig (Fig. 6a-d) was dehydrated in 100% ethanol, immersed in Visikol for a week, washed in 100% ethanol, further cleared in BABB overnight and M-OPT scanned.

### Scanner construction and operation

The specimen is lowered into the cuvette (Fig. 2A), illuminated on one side by a light emitting diode (LED) light array (Fig. 2Aa) and imaged on the other side with a camera attached to a Leica MZFLIII stereo microscope fitted with a 0.32 x achromat objective. The microscope is supported horizontally to achieve a parallel light path through the specimen (Fig. 2A). The specimen is positioned into the focal plane by moving the cuvette and LED light platform, mounted on horizontal runners, towards or away from the microscope (Figs. 2Ac, 3h). The light source for fluorescent illumination for emission M-OPT is an external metal halide light source (EL 6000, Leica Microsystems) coupled to a liquid light guide. The microscope fluorescent filters used are: Texas red (TXR) exciter filter 560/40 nm, barrier filter 610LP to visualise tissue autofluorescence; GFP1 exciter filter 425/60 nm, barrier filter 480 LP and GFP3 exciter filter 470/40 nm, barrier filter 525/50 nm to visualise tissue autofluorescence and green fluorescent protein (GFP) tagged gene expression. The camera is a second generation scientific complementary metal oxide semiconductor (sCMOS) Hamamatsu Orca Flash 4 with 6.5 x 6.5 µm pixel size and 2048 x 2048 pixels sensor array, connected to the microscope via a C-mount adapter (1x delta) and video /photo objective (1x Leica Microsystems) (Fig. 2C). This camera was chosen for its large 4 mega pixels sensor and for its high quantum efficiency (QE) i.e. sensitivity for imaging faint fluorescent light emitted by plants expressing GFP gene activity.

**Fig. 3.**
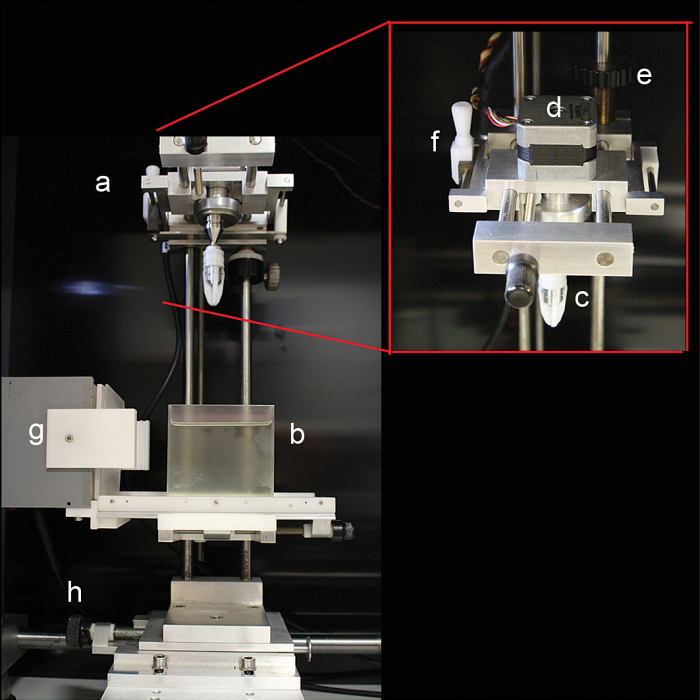
Sample attachment and rotation assembly. (a) Attachment and rotation assembly (b) cuvette (c) grabber (d) motor (e) vertical movement wheel (f) sample alignment bar (g) LED light array (h) focus wheel.

**Fig. 4.**
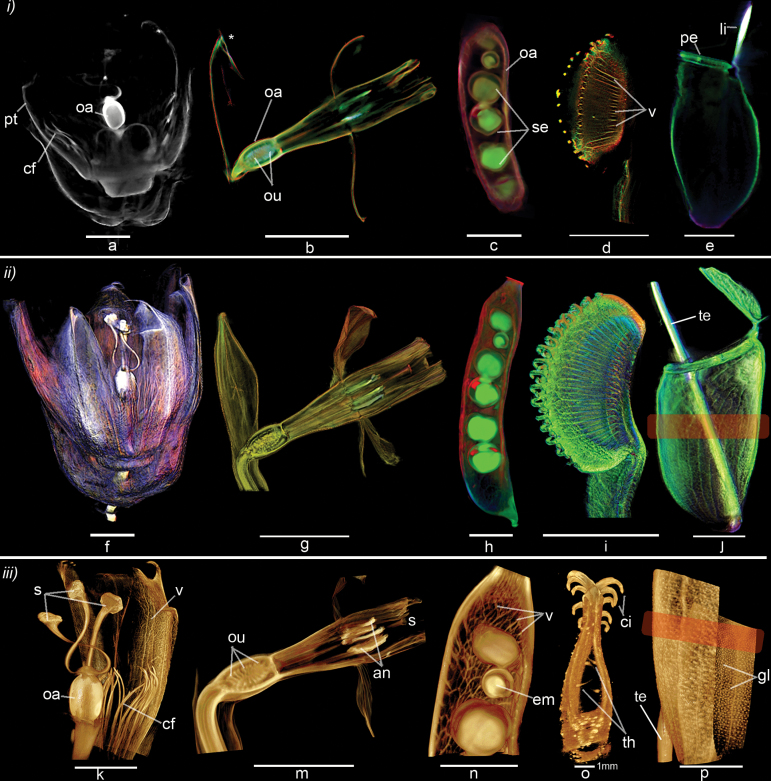
Visualising mature flowers, leaves and fruits up to 60 mm tall and 45 mm deep in 3D with M-OPT. *(i*) M-OPT longitudinal sections illustrating internal details of a range of plant organs. (a) Passion flower *(P. caerulea*) fluorescent emission M-OPT using a GFP1 filter with a scan resolution of 62.5 µm/voxel. cf, coronal filaments; oa, ovary; pt, petal. (b) Daffodil *(N. peoticus*) flower with transmisison (red) plus two fluorescent emission M-OPT channels combined using a TXR filter in green and GFP1 filter in blue, with a scan resolution of 40 µm/voxel. oa, ovaries; ou, ovule. Asterisk indicates a movement defect (c) Pea pod (*P. sativum*) with transmission in red, plus two emission channels using a TXR filter in green and GFP1 filter in blue, with a scan resolution of 62.5 µm/voxel. oa, ovary; se, seed. (d) Immature Venus fly trap (*D. muscipula*) with transmission in red plus an emission channel using a GFP1 filter in green, with a scan resolution of 38.2 µm/voxel. v, veins. (e) *Nepenthes* pitcher leaf trap with transmission in red and fluorescent emission M-OPT channels using a TXR filter in green and GFP1 filter in blue, with a scan resolution of 62.5 µm/voxel. li, lid; pe, peristrome. VolViewer sectional views. Scale bars, 1 cm. (ii) 3D Volume renderings of M-OPT datasets shown in (i). (f) Passion flower volume view (g) Daffodil flower, clipped volume view of transmisison and two emission M-OPT channels combined (h) Pea pod clipped volume to reveal peas inside (i) Immature Venus fly trap volume view showing the arrangement of veins in the valve and interlocking cilia at valve margins. (j) Internal clipped volume view of *Nepenthes* pitcher leaf trap shown in (e). Upper portion of the leaf has a smooth waxy surface. Lower part of the leaf normally contains a pool of digestive enzymes and has a different texture to the upper part of the leaf. The boundary between the two zones is highlighted in orange. The tendril (te) that supports the trap is shown behind the trap structure. VolViewer volume renderings with non-photorealistic illumination applied (f, g, i, j). Scale bars, 1 cm. (iii) Regions of interest in M-OPT datasets shown in (i) and (ii). (k) Passion flower, GFP1 emission channel volume cut to reveal: s, stigmas; oa, ovary; cf, coronal filaments; v, veins, in the petal. (m) Daffodil flower, GFP1 emission channel volume clipped to reveal: ou, ovaries; an, anthers; s, stigma. (n) Pea pod, TXR emission channel volume cut to reveal pea seed with embryo inside. em, embryo; v, veins. (o) Venus fly trap transmission channel clipped volume showing interlocking cilia, trigger hairs and an empty region between the two leaf valves. ci, cilia; th, trigger hairs (p) *Nepenthes* pitcher leaf trap shown in (e, j). The transmission M-OPT channel is cut and rotated to show the front surface of the trap and the inner surface with digestive glands. gl, glands; te, tendril. Drishti volume renderings applied. Scale bars, 1 cm, unless otherwise stated.

Alignment of the camera and specimen is required for good quality reconstructions. The adjustable platform, holding the microscope at 90^o^ to the sample holder mount, is centred and aligned with adjustable bolts to bring the microscope square to the specimen (Fig. 2A). The camera is aligned with the sample using a rotatable gear adapted from a MRC Prototype OPT Scanner (Fig. 2Ca). The camera is controlled by a Micromanager (https://www.micro-manager.org/, last accessed 24 November 2016) plugin for Image J (http://imagej.net/, last accessed 25 November 2016), with additional scripts written to control stepper motor rotation, the fluorescent light source shutter and LED illumination via an electronic control box. The sample attachment and rotation assembly (Fig. 3a) is mounted on vertical runners allowing the specimen to be lowered into the cuvette (Fig. 3b), containing BABB for cleared tissues or water for live plants. The specimen is held at 90° to the light path and centred using a lever to nudge the sample into its centre of rotation (Fig. 3f). When optically zooming in on objects within specimens, for example insects within plant organs (Fig. 6d, g) and stained veins inside a stem (Fig. 7e), the specimen is recentred about that object. Once centred and focussed the sample is rotated through 360° using a stepper motor (Fig. 3d) with 400 images captured at 0.9° steps. Rotation of large fragile structures like petals can cause unwanted movement resulting in misalignments during reconstruction. An example of this is shown at the tip of the sepal of the Daffodil flower (*Narcissus peoticus*) (Fig. 4b). To stabilise specimens after each rotation a pause of 100 ms was introduced before image acquisition. This pause allows any movement in the system to subside and minimises the motion effect in reconstructed scans. The images are post-processed in preparation for reconstruction using OPTNorm scripts (Matlab software, http://uk.mathworks.com/, last accessed 25 November 2016) and reconstructions carried out using NRecon software (Bruker, http://bruker-microct.com/products/downloads.htm, last accessed 25 November 2016).

The reconstructed M-OPT slice images are loaded into VolViewer in 16 bit.tiff format to visualise and quantify the dataset in 3D (Figs. 4i, ii, 5, 6a-d, g, 7f-g) (VolViewer software, http://cmpdartsvr3.cmp.uea.ac.uk/wiki/BanghamLab/index.php/VolViewer, last accessed 25 November 2016) (Lee *et al.*, 2006). Noise is removed and slices converted to 8 bit.png format. Opacity and intensity of the volume is adjusted using the transfer function editor and internal structures within the volumes are interactively explored with the clipping editor. Measurements are made with the VolViewer measuring editor. For some datasets VolViewer is used for cropping the volume with the crop editor, despeckling via the median filter, and application of non-photorealistic lighting with the lighting editor. An alternative 3D visualisation software called Drishti is also used (Figs. 4iii, 6e, f, h-j, 7b-e, h-j) (Limaye, 2012) (https://sf.anu.edu.au/Vizlab/drishti/, last accessed 25 November 2016).

Voxel image scale is calculated based on magnification and verified in M-OPT scans of glass microspheres (210 µm, Whitehouse Scientific) and a reticule (10 mm/ 0.1 mm, Pyser 01B212). Depending on the magnification a spatial resolution of 6.5 µm/voxel to 63 µm/voxel is achieved.

A single OPT channel does not capture all the information available in a specimen. To extract more information, both transmission and fluorescent emission M-OPT scans can be superimposed by sequentially collecting each channel at the same magnification, focus setting and angle (Sharpe *et al.*, 2002; Lee *et al.*, 2006). Using VolViewer, this data can be combined by loading each volume as either a red, green or blue channel into the same space. Image opacity and intensity can be independently adjusted with the transfer function editor. Combining fluorescence emission and transmission M-OPT channels can provide contextual information for gene expression or reveal complementary structural details in the same specimen. An example of combined M-OPT scans is shown for a pea pod *(Pisum sativum*) (Fig. 4c, h). This combination of channels results in the visualisation of additional details that can be missed when imaging a single M-OPT channel. For example, tissue autofluorescence shown in green in the pea pod was collected with fluorescent emission M-OPT using the TXR filter, whereas the carpel wall (red) of the pod was collected with the transmission M-OPT channel (Fig. 4c, h, Supplementary Video S1 available at JXB online). Additionally a second autofluorescence channel visualised with the GFP1 filter (blue) was more intense at the tip of the pod (Fig. 4h). Thus some M-OPT datasets are improved when a combination of channels are viewed together.

**Fig. 5.**
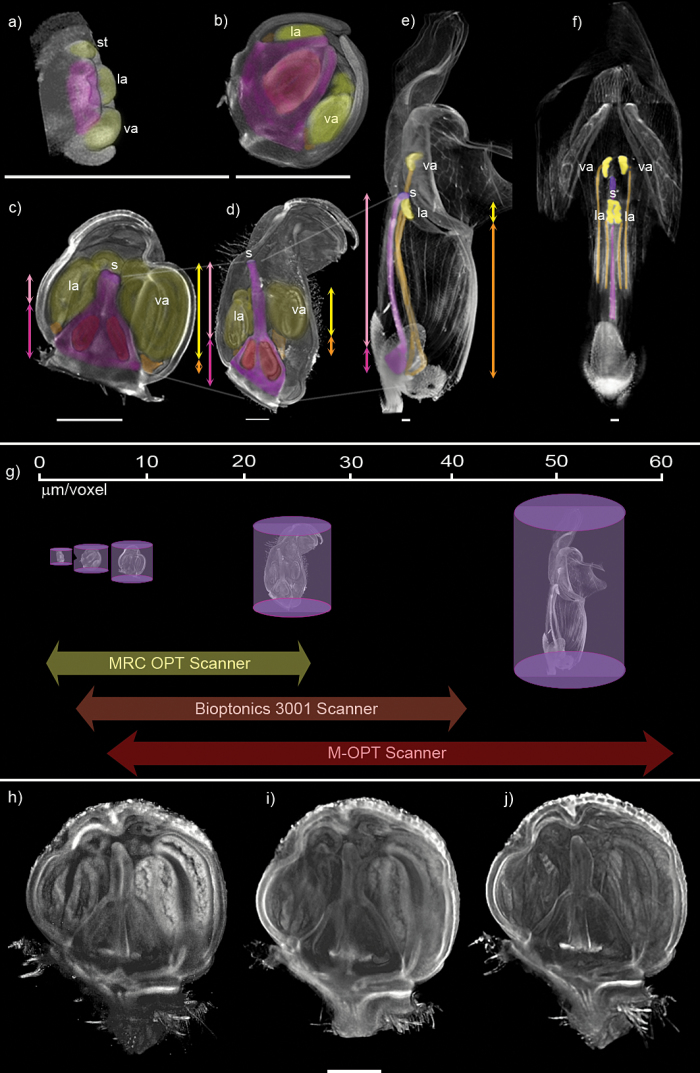
Comparison of OPT systems. (a-f) Comparison of OPT systems illustrating spatial relationships of developing snapdragon floral organs. (a-b) MRC OPT scanner. Snapdragon buds 0.5 mm to 1.2 mm in size were emission OPT scanned using a GFP1 filter. The volume was clipped to reveal internal organs. (a) Gynoecium primordium at the centre of the 0.1 mm tall bud, highlighted in pink, is surrounded by a 0.2 mm tall anther, highlighted in yellow, and petal primordia. Scan resolution of 3 µm/voxel. (b) The gynoecium expanded to 0.7 mm tall and is filled with the ovule domain. Anthers of 0.5 mm in height are supported by short filaments 0.4 mm in length, which are partially visible in this view. Petals have extended to envelop the anthers and gynoecium. Scan resolution of 4.8 µm/voxel. (c-d) Bioptonics 3001 OPT scanner. Buds 2.5 mm to 7 mm deep and 2.6 mm and 10 mm tall were transmission OPT scanned. (c) Ovaries are visible in the gynoecium. The style has elongated to 0.6 mm making the gynoecium 1.5 mm tall from the base of the ovule to the stigma and bringing the stigma level with the top of the anthers, which are supported by short filaments. Both lateral anther filaments are 1.1 mm long. Both ventral anther filaments are 1.3 mm long. Petals form a tube around the gynoecium and anthers, overlapping and folding above. Scan resolution of 8.2 µm/voxel. (d) The petal tube is elongated and is a similar length to the extended style at 3.9 mm, which supports the 1.6 mm long stigma above the pollen containing anthers. The gynoecium from the base of the ovule to the stigma measures 6.1 mm. Lateral anther filaments are 2.8 mm and 3.3 mm long. Ventral anther filaments are 4.2 mm and 4.4 mm long. Petals fold above the tube and floral organs. Scan resolution of 24.4 µm/voxel. (e-f) M-OPT scanner. A mature flower 15 mm deep and 40 mm tall was emission M-OPT scanned using a GFP1 filter. (e) The petal tube has elongated and remains a similar length to the style, which has lengthened considerably to 10.7 mm from the top of the ovule to the stigma. The gynoecium from the base to the stigma measures 24.6 mm. Distally, mature petals have unfolded. Anthers containing the male pollen are supported by much elongated filaments and held at two levels in the flower above and level with the female stigma. Lower ventral anther filaments are 18.2 mm long, upper lateral anther filaments are 20 mm long. (f) Ventral view of clipped volume shown in (e) highlighting the arrangement of the anthers around the stigma in the mature flower. Scan resolution of 50.4 µm/voxel. la, lateral anther; va, ventral anther; s, stigma; st, staminode, a vestigial anther that does not develop further. Gynoecium in pink, ovule in red, stigma in purple, anthers in yellow, and anther filaments in orange. Double ended arrows show the proportion of ovary to stigma in developing carpels; pale pink refers to style height and dark pink to ovary height. Double ended arrows show the proportion of anther to filament in developing flowers; yellow refers to anther height and orange to filament height. OPT scans were volume rendered and clipped with VolViewer software. Scale bars, 1 mm. (g) Cartoon illustrating resolution range. Arrows and images show relative resolution range of OPT Scanners. Scale bar is in µm/voxel. The M-OPT scanner resolution range is from 6.5 µm/voxel to 62.5 µm/voxel, with a maximum sample size of 60 mm tall by 45 mm deep. Images are collected with a Hamamatsu Orca Flash 4 camera via a Leica MZFLIII microscope fitted with a 0.32 x achromat objective, 1x phototube and 1x c-mount adaptor. Different objective lenses could be used for higher resolution imaging with the M-OPT scanner but at the expense of not being able to image larger specimens. The Bioptonics Scanner 3001 resolution range is from, 3.2 µm/voxel to 40 µm/voxel, with a maximum sample size of 15 mm tall by 10 mm deep. The MRC OPT scanner resolution range is from 1.6 µm/voxel to 26.5 µm/voxel, with a maximum sample size of 15 mm tall by 10 mm deep. MRC OPT Scanner images are captured with a Hamamtsu Orca-ER-1394 camera via a Leica MZ16FA microscope fitted with 0.63x PLAN APO Leica objective, 1x phototube and 1x c-mount adaptor. (h-j) Image quality of the M-OPT scanner compared to existing OPT systems. Snapdragon bud emission OPT scanned on the three systems at the same scan resolution of 7.7 µm/voxel. View through the centre of the bud is shown. (h) M-OPT scanner (i) Bioptonics 3001 scanner (j) MRC OPT scanner. Scale bar, 1 mm. Volume rendered with VolViewer software.

**Fig. 6.**
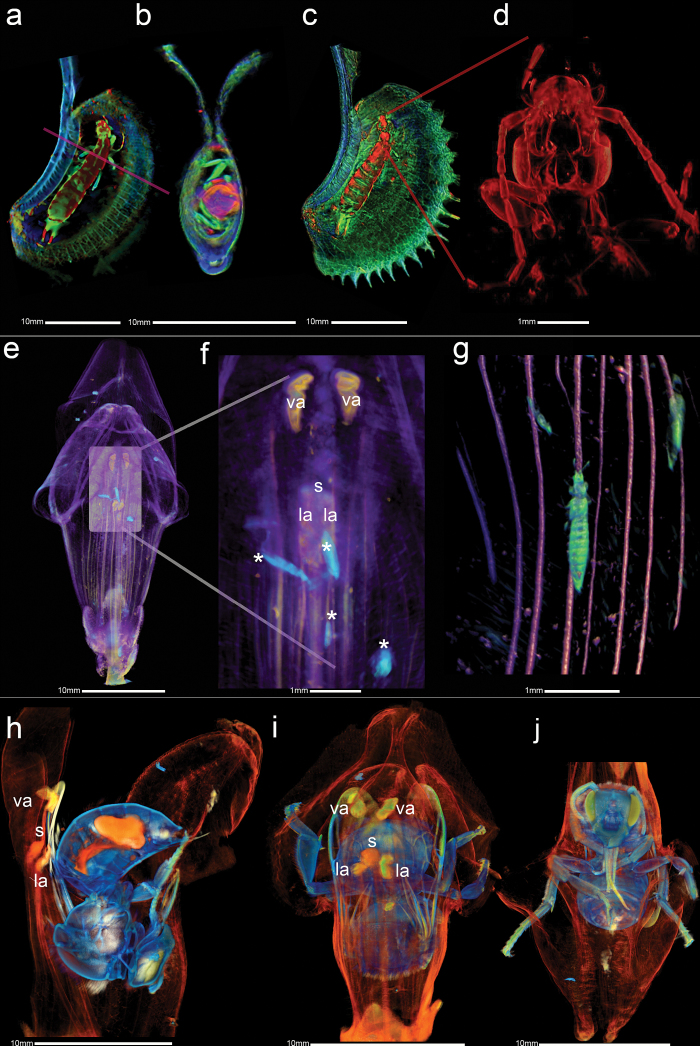
3D biotic relationships between plants and insects. (a-d) Interaction of plant traps with insects. Venus fly trap (*D. muscipula*) and earwig (*F. auricularia*) prey visualised with M-OPT transmission (red) and two fluorescent emission channels, using a TXR filter in green and a GFP1 filter in blue. Combined together this emphasises the different features of the insect and leaf. (a) Section view with combined M-OPT channels. The insect exoskeleton and internal regions of the head and thorax are visible in the transmission M-OPT channel (red), veins in leaf tissue, limbs and segmented regions of the earwig in green show tissue autofluorescence collected via the TXR filter. Another autoflorescence channel collected via the GFP1 filter is shown in blue. The magenta line in (a) shows the location of the cross section (b) through the insect and trap volume view. (c) Clipped longitudinal volume view of insect and trap. Red lines in (c) indicate the region of earwig viewed at maximum M-OPT zoom in (d) single emission M-OPT TXR channel (red). Scan resolutions are 40 µm/voxel (a-c) and 6.5 µm/voxel (d). Renderings were made with VolViewer software. (e-j) Relationships of insects with pollen. (e-g) Thrips (*Thysanoptera*) inside a snapdragon (*A. majus*) flower visualised with M-OPT. (e) Ventral volume view of flower. Two M-OPT channels are combined using Drishti software. Anthers (yellow) imaged using fluorescent emission M-OPT with a GFP1 filter. Petals (purple) and thrips (turquoise) imaged using the transmission M-OPT channel. (f) Zooming in with visualisation software shows the arrangement of thrips in relation to anthers and stigma, with thrips (asterisks) around lateral anthers (la). (g) The same specimen shown in (e-f) M-OPT scanned at maximum zoom, showing thrips in relation to floral veins. Combined transmission (green) and fluorescent emission channel using a GFP1 filter (red), with non-photorealistic illumination applied using VolViewer software. Scan resolutions of 40 µm/voxel (e-f) and 6.5 µm/voxel (g). (h-j) Snapdragon flower *(A. majus*) and pollinating bee *(Apis*) visualised with three M-OPT channels combined. (h) Clipped M-OPT volume view through the centre of a bee inside the flower. The transmission M-OPT channel (orange) highlights petals and internal organs and the exoskeleton of the bee. Tissue autoflorescence collected via the GFP1 filter is shown in yellow/green and was brightest in the insect’s eyes, legs, and wings and flower anthers. Another autoflorescence channel collected via the TXR filter is shown in blue and is most prevalent in the exoskeleton. (i) Dorsal view of the bee inside the flower showing the spatial relationship between the anthers and stigma with the back of the bee. Anthers are in a rectangular conformation with the stigma in the centre. (j) Clipped ventral volume view of the bee inside the flower. Scan resolution of 40 µ/voxel. Drishti volume renderings applied (h-j). va, ventral anther; la, lateral anther; s, stigma; asterisks, thrip.

**Fig. 7.**
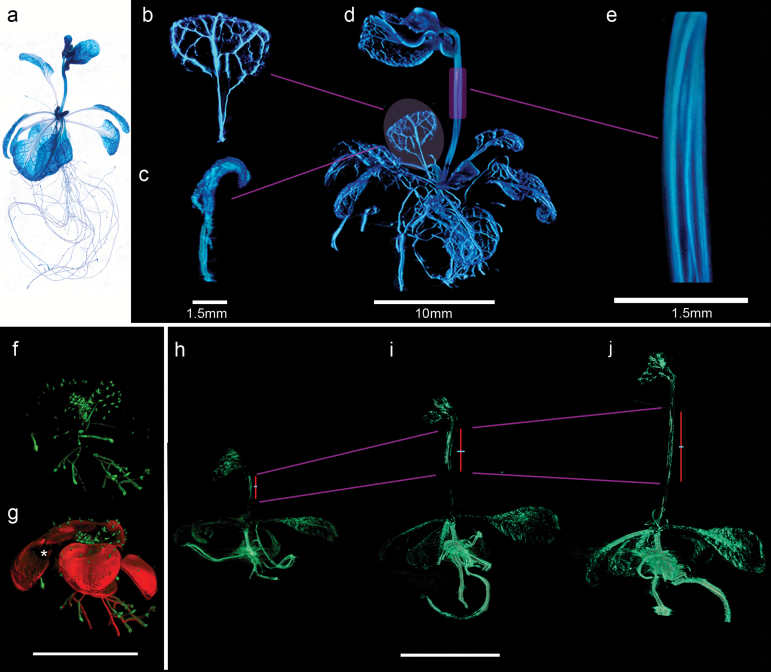
Gene activity in 3D in whole fixed and living Arabidopsis plants. (a-e) Gene expression in a fixed whole Arabidopsis plant. (a) Dissecting microscope image of a plant with AthB8::GUS expression in the veins and roots. (b-d) Transmission M-OPT volume view of a different plant (d) from that shown in (a) at 24 days after sowing, with AthB8::GUS expression and stained with β-Glucuronidase to show gene expression in veins and roots (blue). (b) Detail of veins in leaf virtually dissected from (d), (c) Side view showing leaf curvature. (e) Cropped volume view of stem from the plant shown in (d) scanned at a higher magnification and showing twisting of the veins. Scan resolutions of 24.9 µm/voxel (b-d) and 6.47 µm/voxel (e). Scale bar, 1.5 mm. Drishti volume renderings applied. (f, g) Gene expression in a whole live Arabidopsis plant. Living transgenic Arabidopsis plant with glabra 2::GFP fluorescence visualised with M-OPT at 19 days after sowing. (f) Volume view of a living plant imaged by emission M-OPT using the GFP3 filter shows trichome hairs on the leaf surface in green. (g) Multi-channel volume rendering with transmission M-OPT channel for plant structure (red) superimposed on the emission M-OPT channel (green). Asterisk denotes occlusion holes in the transmission channel volume view. Scan resolution of 29.8 µm/voxel. Scale bar, 10 mm. VolView volume renderings applied. (h-j) Visualising gene activity in 3D in a whole growing Arabidopsis plant. (h) A transgenic Arabidopsis plant 27 days after sowing, expressing GFP clonal sectors after a 3.5 minute heat shock induction, which was embedded in agarose 7 days after treatment. Emission M-OPT images collected via the GFP3 filter. After imaging the plant was returned to normal growth conditions before embedding in fresh agarose and imaging again the following day (i). This was repeated again 24 hours later (j). GFP gene activity shown in green. The sector region highlights faster growth parallel to the stem (red line). Growth perpendicular to the stem is lower (turquoise line). Scan resolutions of 19.7 µm/voxel (h), 24.9 µm/voxel (i) and 30 µm/voxel (j). Scale bar, 10 mm. Drishti volume renderings applied.

For figures, images are exported from VolViewer and Drishti and constructed with Adobe Photoshop (Figs. 4–7). For supplementary videos, images are exported from VolViewer and Drishti and animated with Adobe Premier (Supplementary Videos S1–S11).

### Plant and insect materials

Arabidopsis seeds are surface sterilised, stratified for 4 days and grown on plates containing Murashige Skoog (MS) media in a controlled environment room at 20 ^o^C with 16 hours of light and 8 hours of dark. The GUS reporter gene, *uidA*, encoding *Escherichia coli* β-Glucuronidase (Jefferson *et al*., 1987) is used for histochemical localization of gene activity in ATHB8::GUS transgenic Arabidopsis plants of the Columbia ecotype (Kang *et al.*, 2003), acquired from Thomas Berleth. Before GUS staining plants are fixed in acetone for 20 minutes. After staining plants are dehydrated in methanol for a week and cleared in BABB before mounting in the grabber for M-OPT scanning. Staining protocol and preparation for OPT are as described (Lee *et al.*, 2006).

For live imaging of gene activity in Arabidopsis, the GL2::GFP-ER line of the Landsberg erecta ecotype (Lin and Schiefelbein, 2001) is used, acquired from Martin Hülskamp. Plants are pre-screened for GFP fluorescence with a Leica Fluo III stereo microscope, before embedding in agarose and M-OPT imaging in water, without clearing. Arabidopsis cell lineage sectors expressing GFP are generated by heat shocking plants containing 35S-lox-GUS-GFP in a HS::CRE construct background, by administering a 3.5 minute treatment in a 39 ^o^C water bath to induce GFP expression (Gallois *et al.*, 2002; Kuchen *et al.*, 2012). To allow cell lineages to form, plants are returned to the growth room for 3–6 days before M-OPT imaging. For growth snapshots a plant is embedded in 1% low melting point agarose, M-OPT scanned in water, then removed from the agarose, rinsed in sterile water and returned to MS media in the controlled environment room to grow overnight before re-embedding and further scanning.

For M-OPT of a Snapdragon (*Antirrhinum majus*) flower containing a bee (*Apis* genus, species unknown), following entry of a bee, a flower was placed in chloroform. The specimen was dehydrated in methanol for one week and cleared in BABB for approximately one year before M-OPT scanning. A garden grown Venus fly trap *Dionaea* muscipula ‘saw-tooth’ with a closed trap containing an earwig (*Forficula auricularia*) was placed in 100% ethanol overnight and cleared in Visikol (Villani *et al.*, 2013) for one week before rinsing in 100% ethanol and M-OPT scanning in BABB.

Other plant tissues used include Snapdragon *Antirrhinum majus* var. *striatum* (collected from La Molina, Spain), garden grown passion flower *Passiflora caerulea*, pea pod *Pisum sativum*, monkey cup pitcher plant *Nepenthes ventricosa x inermis* (Hampshire Carnivorous Plants), garden grown daffodil *Narcissus* peoticus ‘Tete a tete’ and *Dionaea muscipula* (Hampshire Carnivorous Plants). Plant organs including leaves, flowers and fruits, are dehydrated in methanol for a week and cleared in BABB before mounting in the grabber for M-OPT scanning.

## Results

### Visualising mature flowers, fruits and leaves up to 60 mm in length

Existing OPT scanners preclude 3D visualisation of larger tissues. To extend specimen size the M-OPT scanner was custom built, taking advantage of improvements in sample handling and camera technology, using a light sensitive second generation sCMOS camera. This configuration of microscope, camera and supporting framework could be readily replicated in other labs with access to a workshop. The combination of a large quartz cuvette and sample grabber has simplified preparation, making it possible to use large specimens. M-OPT can accommodate a large range of sample sizes with resolutions ranging from 6.5 µm/voxel to 62.5 µm/voxel. One of the largest specimens visualised was a Passion flower (*P. caerulea*) that was 60 mm long and 45 mm deep (Fig. 4a, f, k, Supplementary Video S2). Rather than physically sectioning the specimen with a blade, OPT allows virtual optical sectioning, illustrated here with a slice through the centre of the flower revealing the petals (pt), corona filaments (cf) and ovary (oa) (Fig. 4a). When virtual optical sections of the whole M-OPT dataset are viewed together as a volume the overall 3D shape is a good representation of the flower (Fig. 4f, Supplementary Video S2). Clipping the volume reveals details such as petal venation (v) and the relative spatial organisation of the ovary (oa), stigmas (s) and coronal filaments (cf) to each other. Three stigmas are supported by curved styles above a single ovary. The carpel is surrounded by a whorl of coronal filaments, in turn surrounded by an outer whorl of petals (Fig. 4k). Thus, with M-OPT it is possible to obtain 3D images from plant organs previously too large to be imaged by other OPT systems.

The virtual section through the passion flower did not show internal details such as developing ovules within the ovary due to insufficient optical clearing (Fig. 4a). Such specimens may benefit from treatment with novel clearing agents (Villani *et al.*, 2013; Kurihara *et al.*, 2015; Palmer *et al.*, 2015). However these structures could be visualised in other species that were easier to clear. For example, a daffodil flower with developing ovules (ou) 0.4 mm in length within a 5.5 mm long ovary (oa) were visualised at the base of the flower (Fig. 4b, m, Supplementary Video S3). The daffodil flower has a single style and stigma (s) in the carpel (Fig. 4m) while the passion flower has three styles (s) (Fig. 4k). Thus, internal structures of flowers, such as carpels can be visualised by M-OPT and compared to other species.

The visualisation of carpel structures in the passion flower and daffodil does not illlustrate later stages of seed development. To observe developing seeds in a mature ovary M-OPT images were collected from a pea pod that was 58.4 mm tall and 10 mm deep. The 3D arrangement of seeds inside the fruit were observed (Fig. 4c, h, Supplementary Video S1). The pea carpel consists of an expanded ovary (oa) surrounding the ovules that have been pollinated to become seeds (se). Remnants of the stigma and style remain at the tip. Virtual dissection of the volume reveals the embryo (em) developing inside the seed and intricate venation (v) patterns in the ovary wall (Fig. 4n). Thus, M-OPT allows observation of 3D shape and texture in both internal and external regions in a fruit.

In addition to flowers, M-OPT was used to obtain 3D images of the complex leaves of plants adapted to capture insect prey, for example *Dionaea muscipula*, the Venus fly trap. The *Dionaea* leaf consists of two halves or valves that rapidly close to capture insects (Fig. 4d, i, o, Supplementary Video S4). The sectional view highlights veins (v) within a single valve of an immature leaf trap (Fig. 4d). Clipped volume views show interlocking cilia (ci) where the two valves come together at the leaf margins (Fig. 4i). In the closed trap the valves curve inwardly with a concave shape creating a sealed inner space, inside which the trigger hairs (th) can be observed (Fig. 4o). 3D visualisation of leaf shape and curvature, in combination with features such as trigger hairs and interlocking cilia at the valve margins, provides an insight into the spatial relations of trapping features in *Dionaea* leaves.

Carnivorous plants have developed an array of leaf shapes as adaptions to trap prey. Another example presented here is the pitcher leaf trap of *Nepenthes ventricosa x inermis*, which was 49.3 mm tall and 17.5 mm deep. Previously this specimen would have been too large to be imaged in existing OPT scanners. The M-OPT sectional view shows the cupped shaped outline of the leaf with a lid (li) and peristome (pe) at the top (Fig. 4e). This leaf acts as a pitfall trap. Insects are attracted by nectar secreted at the peristome and fall into a pool of digestive juices secreted at the base of the trap. Combined sections viewed as a volume show the inside of the trap and the boundary domain between the waxy top part of the pitcher and the bottom (orange rectangle) (Fig. 4j). When virtually dissected, the arrangement of secretary glands (gl) at the bottom of the trap could be observed (Fig. 4p). Thus M-OPT can be used to view the spatial arrangement of functional regions within diverse leaf types.

### Complementing existing OPT systems using M-OPT

To illustrate how M-OPT complements existing OPT scanners we obtained 3D snapshots from a range of machines of a series of developing snapdragon buds and flowers (Fig.5, Supplementary Video S5). The smallest buds were imaged with a MRC OPT scanner (Fig. 5a, b). 3D datasets of larger buds were collected with a Bioptonics Scanner 3001 (Fig. 5c, d) and the mature flower visualised with M-OPT (Fig. 5e, f). A cartoon illustrates scanner resolution in relation to bud development (Fig. 5g). The M-OPT scanner with it’s current lens arrangement cannot achieve the highest resolution possible with the MRC OPT scanner. However, in this format M-OPT covers most of the range of the MRC scanner and Bioptonics Scanner 3001 and extends the range to larger specimens. Additionally, the quality of images obtained with the M-OPT scanner (Fig. 5h) are comparable to those obtained with the Bioptonics 3001 OPT scanner (Fig. 5i) and MRC OPT scanner (Fig. 5j).

Without M-OPT, the largest image would have been the immature bud shown in Fig. 5d. At this stage the style is about twice the length of the ovary and the lateral (la) and ventral anthers (va) lie at a similar level to each other below the stigma (s). By having the M-OPT image, we can see how these relations change as the flower matures (Fig. 5e). In the mature flower the style is six times longer than the ovary, showing that it grew at a faster rate. Similarly, the stamen filaments have greatly extended, raising the anthers so that the two lower lateral anthers (la) lie slightly below the stigma (s) and the two higher ventral anthers (va) are above the stigma in a rectangular conformation (Fig. 5f). The growth of these structures can be traced back using higher resolution OPT to earlier stages (Fig. 5a-d). Ventral anthers (va), located at the bottom of the youngest buds, overtake lateral anthers (la) to be at the top of the mature flower due to faster growth of the ventral anther filaments (see yellow and orange arrows in Fig. 5c, d, e). The carpel differentiates into the clearly defined structures of ovary, style and stigma, which change in relative lengths (see pink and magenta arrows in Fig. 5c, d, e). Thus, M-OPT allows 3D imaging of the developmental stages of a Snapdragon flower to be completed, revealing how certain regions of an organ can grow differently relative to other regions.

### Visualising plant-insect interactions in 3D

Previous M-OPT images show architectural details of individual organs and the changing spatial relationships between organs in a developing flower (Figs. 4, 5). Plants can also have complex biotic interactions where prey, parasites or pollinators are hidden within them. To observe such interactions in 3D, M-OPT was applied to a variety of plant leaf traps and flowers containing insects (Fig. 6).

The Venus fly trap captures insects to obtain nutrients (Fig.4). M-OPT images were collected of a Sawtooth *D. muscipula* trap containing prey and revealed a captured earwig (*Forficula auricularia*) (Fig. 6a-d, Supplementary Video S6). A view of a longitudinal section shows the earwig positioned in the trap close to the midrib (Fig. 6a). The earwig is 17 mm long and spans almost the entire length of the trap, taking up approximately half of the depth of the leaf. The earwig is surrounded by a small region of empty space also visible when the earwig and trap are viewed in cross section (Fig. 6b). At maximum resolution M-OPT makes it possible to zoom into 3D structural details of the earwig inside the trap (Fig. 6d). Thus, M-OPT allows the identification of insects trapped by carnivorous plants and the positioning of prey to be viewed together in relation to leaf structure.

The Sawtooth *Dionaea* is a mutant strain with wider than normal teeth, known as cilia, at the trap margins; these cilia do not interlock (Fig. 6b, c). Instead the leaf itself forms a tight seal around the prey whilst the marginal portion of the trap remains open (Fig. 6b). By contrast, a normal trap has interlocking cilia (ci), creating a barrier to escape (Fig. 4o). The Sawtooth trap (Fig. 6a-c) has nonetheless maintained an effective trapping mechanism even though the cilia do not interlock as they do in the wild type. Thus, M-OPT allows leaf trap shape in morphologically different Sawtooth and normal Venus fly trap strains to be compared in 3D, providing insights into trapping mechanisms and interactions of plants with their prey.

A M-OPT dataset of a mature Snapdragon flower (Fig. 5e, f) on closer examination was also found to contain insects. The M-OPT volume view shows a population of thrips (*Thysanoptera*) located close to the anthers (Fig. 6e-g, Supplementary Video S7). Thrips are 1 mm long compared with anthers which are 2 mm long. Thrips feed on pollen, reducing the fertility of the flower and can also carry viruses that adversely affect the plant. M-OPT can be used to find insects and observe their spatial relationships with plant organs in 3D, providing insights into plant interactions with insect populations.

To further illustrate the potential of using M-OPT to visualise insect-plant interactions, 3D images of a bee (*Apis*) inside a Snapdragon flower were obtained. The lower lip of the flower comprises united lateral and ventral petals that act as a pollinator landing pad. For comparison, a closed flower is shown in Fig. 5e. A pollinator pushes the flower open as it enters head first, lured by nectar at the base. The longitudinal clipped volume shows the 28 mm long flower in the open position with a 12 mm long bee inside (Fig. 6h, Supplementary Video S8). Two thirds of the length of the flower is occupied by the bee. Two lateral and two ventral anthers (la, va, respectively) are dorsally placed on flexible elongated filaments arranged in a rectangular conformation. This conformation is preserved when a pollinator is present in the flower and so deposits pollen on the dorsal surface of the bee in four places (Fig. 6h, i). The stigma (s) at the centre of the anther (la, va) arrangement also comes in contact with the back of the bee, making it possible for the flower to receive pollen from visits to previous flowers. It is possible that the insect could move during mounting and M-OPT scanning and so this may not reliably reflect the *in vivo* positioning of the insect within the plant. However, although the posture, positioning and interaction may not be the same as a living bee, the relationship between bee, flower opening and pollination are captured together with M-OPT. Thus M-OPT allows alterations in flower shape and interactions of floral organs with a pollinator to be visualised in 3D.

### Visualising gene activity in 3D in fixed and living plants

The ability to visualise gene expression in whole plants is important for understanding gene activity during development. We tested whether M-OPT allows visualisation of reporter gene expression in both fixed and living whole Arabidopsis plants (Fig. 7). In fixed transgenic plants containing the *Arabidopsis thaliana* homeobox gene 8::beta-glucuronidase (AthB8::GUS), histochemical staining highlights veins within tissues and organs, reflecting AthB8 promoter activity (Fig. 7a). After staining, plants were cleared and M-OPT images collected. The resulting volume views show the intricate venation pattern in the leaves (Fig. 7b-d, Supplementary Video S9). Zooming into the stem by imaging with M-OPT at higher resolution shows the spacing of the veins in relation to each other (Fig. 7e, Supplementary Video S9). 3D visualisation of AthB8::GUS expression in the veins reveals twisting of the stem in the bolting Arabidopsis plant. M-OPT thus enables gene expression in the context of an entire plant to be observed in 3D and can also reveal internal features.

To explore the potential of M-OPT in live imaging gene expression, transgenic Arabidopsis plants expressing a glabra2::GFP construct were embedded in agarose and emission M-OPT scanned in water to capture the GFP fluorescence of trichomes (Fig. 7f, Supplementary Video S10). The volume view shows GFP fluorescence in the trichome hairs on the leaf surfaces and in the roots. Superimposing the transmission M-OPT channel with the emission M-OPT channel provides details of the surrounding tissues, shown in red (Fig. 7g). The occlusion of light through overlaying leaves in these uncleared plants causes ‘empty’ regions and artefacts in the reconstruction. However, even incomplete leaf outlines combined with the observation of gene expression provided by live M-OPT allows 3D visualisation of gene activity in the context of the whole living plant.

It is important not only to consider changing tissue shape but also to be able to observe gene activity over time within growing tissues and organs. M-OPT was used to view fluorescent epidermal sectors, generated by heat shock induction of 35S-lox-GUS-GFP in a HS::CRE construct background, in the same plant imaged at daily intervals over a period of 3 days. Elongating GFP sectors in the stem indicate growth is greater parallel to the growing stem than perpendicular. The sector region increases in length by 39% between day 1 and 2 (Fig. 7h, i) and by 56% between days 2 and 3 (Fig. 7i, j), while it increases in width by 9% between day 1 and 2 (Fig. 7h, i) and by 8% between days 2 and 3 (Fig. 7i, j). Embedding the plant in agarose could mechanically constrain growth, however the period over which scans are collected is brief and embedding is thought to have little effect on overall growth. M-OPT thus enables imaging of surface GFP fluorescence in leaf hairs and epidermal sectors in leaves, flowers, stems and roots, which allows tracking of gene activity in whole growing and flowering Arabidopsis plants.

## Discussion

We show how M-OPT can be used to visualise 3D morphology and follow patterns of gene expression in whole growing plants. Confocal and light sheet microscopy are widely used to visualise gene expression with fluorescent labels at cellular and subcellular levels (Bassel and Smith, 2016). M-OPT allows the observation of gene expression in growing plants at the whole organ and plant scale, using fluorescently marked surface features including leaf trichome hairs and epidermal sectors, as well as in semi-transparent roots. Further work is required to determine the depth to which fluorescent markers such as GFP can be reliably detected inside uncleared living tissues with M-OPT. Previously clonal sectors or tracking have been used to follow growth in single organs (Kuchen *et al.*, 2012; Tauriello *et al.*, 2015; Hervieux *et al.*, 2016). In future M-OPT could be applied to visualising growth using clonal sectors as markers in whole plants in 3D.

Imaging of developing tissues, using either HRCT in whole Arabidopsis rosettes or confocal optical sectioning in young single Arabidopsis leaves, has been used to take 3D snapshots of different staged leaves (Kaminuma *et al.*, 2008; Failmezger *et al.*, 2013). With M-OPT, trichome patterning could be visualised live and be used to track leaves on a whole plant. Segmentation and quantification tools, such as those illustrated in the 4D confocal imaging of trichome development in young *Capsella* leaves (Barbier de Reuille *et al.*, 2015), could be applied to M-OPT datasets to extract trichome patterning and growth information. M-OPT would also enable visualisation of trichome patterning in larger and higher order leaves, which are distinct from juvenile leaves, exemplifying heteroblasty. For example, the first Arabidopsis leaves to emerge have trichomes only on the adaxial surface, whereas later leaves have trichomes on both abaxial and adaxial surfaces (Telfer *et al.*, 1997). Additionally, leaves become sequentially more dissected in *Cardamine hirsute* (Canales *et al*., 2010). A future application of M-OPT could be the 3D exploration of the hetroblasty of trichome pattering in *Arabidopsis* and leaf dissection in *Cardamine* in whole plants.

Leaf venation patterns also exhibit heteroblasty. For example, AthB8::GUS stained veins, imaged by light microscopy, have been used to explore patterning hierarchy as vein networks become more complex in older leaves (Kang and Dengler, 2004; Alonso-Peral *et al.*, 2006). M-OPT has the advantage, compared to confocal microscopy and light sheet microscopy, that non-fluorescent histochemical stains such as GUS can be visualised in 3D in whole fixed Arabidopsis plants. This could be applied to the study of vein patterning heteroblasty. Additionally, leaf venation of the stem could be used as a marker to study stem twisting or torsion with M-OPT. Macro photography and scanning electron microscopy have been used to compare surface features of the stem in wild type and twisting mutants (Landrein *et al.*, 2013). M-OPT provides both surface and internal views of histochemically marked structures.

In addition to visualising gene expression, M-OPT can be used to reveal internal structures of diverse plant organs previously too deep to be imaged in 3D. When applied to deep plant specimens, both M-OPT and HRCT overcome the maximum size limitation of conventional OPT; however HRTC is relatively underused in plants due to low X-ray absorption making internal structures difficult to visualise (Stuppy *et al.*, 2003; Staedler *et al.*, 2013). We found 3D visualisations of internal structures imaged with M-OPT to be equivalent or better than those obtained with HRCT.

Previously, 3D morphometric studies of flowers imaged with HRCT focussed on measuring corolla shape variation without taking into account stamen and carpel morphology (van der Niet *et al.*, 2010; Wang *et al*., 2015). M-OPT could be used to extend these analyses, quantifying 3D variations in petal shape in concert with carpel and stamen structure, both between species and throughout floral development. Moreover morphometric studies could be related to gene expression using M-OPT.

HRCT has been applied to examine plant-insect interactions between root weevil larvae and clover roots in soil (Johnson *et al.*, 2004) but 3D visualisation of insects within plant organs have not been reported to date. The ability to view internal structures at high resolution in large structures with M-OPT allows biotic relationships of insects with plants, whether parasites, pollinators or prey, to be observed together for the first time. In future M-OPT could be applied to study these interrelationships in more detail. For example, the hunting cycle of the Venus fly trap has been explored (Volkov *et al.*, 2011). M-OPT could be used to extend these studies by visualising how a *Dionaea* leaf deforms in 3D around a trapped insect and whether points of contact between leaf and prey decrease over time as the insect is digested. Fruits or flowers suspected of harbouring insects such as thrips could be imaged by M-OPT, thus providing information about their lifecycle and feeding habits. 3D morphometric studies of corolla shape (van der Niet *et al.*, 2010; Wang *et al*., 2015) could also be extended to look at corolla shape deformations caused by pollinators. It would also be possible to visualise and compare the 3D positioning of stamens and carpels in relation to the pollinating insect using M-OPT.

M-OPT therefore provides an effective 3D imaging platform that allows the study of gene activity, internal plant structures and plant-insect interactions to be extended to a macroscopic scale.

## Supplementary Material

Supplementary Data

## Abbreviations:

AthB8::GUS: *Arabidopsis thaliana homeobox* gene 8;
BABB: 2-parts benzyl benzoate/ 1-part benzyl alcohol;
GFP: green fluorescent protein;
GUS: β-glucuronidase reporter gene;
HRCT: high resolution X-ray computed tomography;
LED: light emitting diode;
M-OPT: macro optical projection tomography;
MRC: Medical Research Council;
MS: Murashige Skoog;
OPT: optical projection tomography;
sCMOS: scientific complementary metal oxide semiconductor;
TXR: Texas red

## References

[CIT0001] Alonso-Peral MM Candela H del Pozo JC Martínez-Laborda A Ponce MR Micol JL 2006 The HVE/CAND1 gene is required for the early patterning of leaf venation in Arabidopsis. Development (Cambridge, England) 133, 3755–3766. 10.1242/dev.02554 16943276

[CIT0002] Arranz A Dong D Zhu S Rudin M Tsatsanis C Tian J Ripoll J 2013 Helical optical projection tomography. Optics Express 21, 25912–25925. 24216818 10.1364/OE.21.025912

[CIT0003] Arranz A Dong D Zhu S Savakis C Tian J Ripoll J 2014 In-vivo optical tomography of small scattering specimens: time-lapse 3D imaging of the head eversion process in Drosophila melanogaster. Scientific Reports 4, 7325. 25471694 10.1038/srep07325 PMC4255187

[CIT0004] Barbier de Reuille P Routier-Kierzkowska A-L Kierzkowski D 2015 MorphoGraphX: A platform for quantifying morphogenesis in 4D. eLife 4, e05864. 10.7554/eLife.05864 PMC4421794 25946108

[CIT0005] Bassel GW Smith RS 2016 Quantifying morphogenesis in plants in 4D. Current Opinion in Plant Biology 29, 87–94. 26748353 10.1016/j.pbi.2015.11.005

[CIT0006] Boot MJ Westerberg CH Sanz-Ezquerro J Cotterell J Schweitzer R Torres M Sharpe J 2008 In vitro whole-organ imaging: 4D quantification of growing mouse limb buds. Nature Methods 5, 609–612. 18516047 10.1038/nmeth.1219

[CIT0007] Borisjuk L Rolletschek H Neuberger T 2012 Surveying the plant’s world by magnetic resonance imaging. The Plant Journal: for Cell and Molecular Biology 70, 129–146. 22449048 10.1111/j.1365-313X.2012.04927.x

[CIT0008] Canales C Barkoulas M Galinha C Tsiantis M 2010 Weeds of change: Cardamine hirsuta as a new model system for studying dissected leaf development. Journal of Plant Research 123, 25–33. 19821009 10.1007/s10265-009-0263-3

[CIT0009] Chen L Andrews N Kumar S Frankel P McGinty J French PM 2013 Simultaneous angular multiplexing optical projection tomography at shifted focal planes. Optics Letters 38, 851–853. 23503237 10.1364/OL.38.000851

[CIT0010] Colas JF Sharpe J 2009 Live optical projection tomography. Organogenesis 5, 211–216. 20539740 10.4161/org.5.4.10426 PMC2878749

[CIT0011] Dhondt S Vanhaeren H Van Loo D Cnudde V Inzé D 2010 Plant structure visualization by high-resolution X-ray computed tomography. Trends in Plant Science 15, 419–422. 20542721 10.1016/j.tplants.2010.05.002

[CIT0012] Dorca-Fornell C Pajor R Lehmeier C 2013 Increased leaf mesophyll porosity following transient retinoblastoma-related protein silencing is revealed by microcomputed tomography imaging and leads to a system-level physiological response to the altered cell division pattern. Plant Journal 76, 914–929. 24118480 10.1111/tpj.12342 PMC4282533

[CIT0013] Failmezger H Jaegle B Schrader A Hülskamp M Tresch A 2013 Semi-automated 3D leaf reconstruction and analysis of trichome patterning from light microscopic images. PLoS Computational Biology 9, e1003029. 23637587 10.1371/journal.pcbi.1003029 PMC3630213

[CIT0014] Fauver M Seibel E Rahn JR Meyer M Patten F Neumann T Nelson A 2005 Three-dimensional imaging of single isolated cell nuclei using optical projection tomography. Optics Express 13, 4210–4223. 19495335 10.1364/opex.13.004210

[CIT0015] Fei P Yu Z Wang X Lu PJ Fu Y He Z Xiong J Huang Y 2012 High dynamic range optical projection tomography (HDR-OPT). Optics Express 20, 8824–8836. 22513593 10.1364/OE.20.008824

[CIT0016] Flavel RJ Guppy CN Tighe M Watt M McNeill A Young IM 2012 Non-destructive quantification of cereal roots in soil using high-resolution X-ray tomography. Journal of Experimental Botany 63, 2503–2511. 22271595 10.1093/jxb/err421

[CIT0017] Gallois JL Woodward C Reddy GV Sablowski R 2002 Combined SHOOT MERISTEMLESS and WUSCHEL trigger ectopic organogenesis in Arabidopsis. Development (Cambridge, England) 129, 3207–3217. 10.1242/dev.129.13.3207 12070095

[CIT0018] Gualda EJ Vale T Almada P Feijó JA Martins GG Moreno N 2013 OpenSpinMicroscopy: an open-source integrated microscopy platform. Nature Methods 10, 599–600. 23749300 10.1038/nmeth.2508

[CIT0019] Hervieux N Dumond M Sapala A 2016 A mechanical feedback restricts sepal growth and shape in Arabidopsis. Current Biology 26, 1019–1028. 10.1016/j.cub.2016.03.004 27151660

[CIT0019a] Hou PW Hsu HC Lin YW Tang NY Cheng CY Hsieh CL 2015 The History, Mechanism, and Clinical Application of Auricular Therapy in Traditional Chinese Medicine. Evidence-based Complementary and Alternative Medicine: eCAM 2015, 495684. 26823672 10.1155/2015/495684 PMC4707384

[CIT0020] Jefferson RA Kavanagh TA Bevan MW 1987 GUS fusions: beta-glucuronidase as a sensitive and versatile gene fusion marker in higher plants. The EMBO journal 6, 3901–3907. 3327686 10.1002/j.1460-2075.1987.tb02730.x PMC553867

[CIT0021] Johnson SN Read DB Gregory PJ 2004 Tracking larval insect movement within soil using high resolution X-ray microtomography. Ecological Entomology 29, 117–122.

[CIT0022] Kaminuma E Yoshizumi T Wada T Matsui M Toyoda T 2008 Quantitative analysis of heterogeneous spatial distribution of Arabidopsis leaf trichomes using micro X-ray computed tomography. The Plant Journal: for Cell and Molecular Biology 56, 470–482. 18643999 10.1111/j.1365-313X.2008.03609.x

[CIT0023] Kang J Dengler N 2004 Vein pattern development in adult leaves of *Arabidopsis thaliana*. International Journal of Plant Sciences 165, 231–242.

[CIT0024] Kang J Tang J Donnelly P Dengler N 2003 Primary vascular pattern and expression of ATHB-8 in shoots of Arabidopsis. New Phytologist 158, 443–454. 10.1046/j.1469-8137.2003.00769.x 36056517

[CIT0025] Kenouche S Perrier M Bertin N 2014 In vivo quantitative NMR imaging of fruit tissues during growth using Spoiled Gradient Echo sequence. Magnetic Resonance Imaging 32, 1418–1427. 25131625 10.1016/j.mri.2014.08.005

[CIT0026] Kuchen EE Fox S Barbier de Reuille P 2012 Generation of leaf shape through early patterns of growth and tissue polarity. Science 335, 1092–1096. 22383846 10.1126/science.1214678

[CIT0027] Kurihara D Mizuta Y Sato Y Higashiyama T 2015 ClearSee: a rapid optical clearing reagent for whole-plant fluorescence imaging. Development (Cambridge, England) 142, 4168–4179. 10.1242/dev.127613 PMC4712841 26493404

[CIT0028] Landrein B Lathe R Bringmann M Vouillot C Ivakov A Boudaoud A Persson S Hamant O 2013 Impaired cellulose synthase guidance leads to stem torsion and twists phyllotactic patterns in Arabidopsis. Current Biology: CB 23, 895–900. 23623553 10.1016/j.cub.2013.04.013

[CIT0029] Lee K Avondo J Morrison H Blot L Stark M Sharpe J Bangham A Coen E 2006 Visualizing plant development and gene expression in three dimensions using optical projection tomography. The Plant Cell 18, 2145–2156. 16905654 10.1105/tpc.106.043042 PMC1560903

[CIT0030] Limaye A 2012 Drishti: a volume exploration and presentation tool. In Proceedings of SPIE Developments in X-Ray Tomography VIII 8506, 85060X–85060X-9.

[CIT0031] Lin Y Schiefelbein J 2001 Embryonic control of epidermal cell patterning in the root and hypocotyl of Arabidopsis. Development (Cambridge, England) 128, 3697–3705. 10.1242/dev.128.19.3697 11585796

[CIT0032] Mayer J Robert-Moreno A Danuser R Stein JV Sharpe J Swoger J 2014 OPTiSPIM: integrating optical projection tomography in light sheet microscopy extends specimen characterization to nonfluorescent contrasts. Optics Letters 39, 1053–1056. 24562276 10.1364/OL.39.001053

[CIT0033] Melkus G Rolletschek H Radchuk R Fuchs J Rutten T Wobus U Altmann T Jakob P Borisjuk L 2009 The metabolic role of the legume endosperm: a noninvasive imaging study. Plant Physiology 151, 1139–1154. 19748915 10.1104/pp.109.143974 PMC2773074

[CIT0034] Metzner R Eggert A van Dusschoten D Pflugfelder D Gerth S Schurr U Uhlmann N Jahnke S 2015 Direct comparison of MRI and X-ray CT technologies for 3D imaging of root systems in soil: potential and challenges for root trait quantification. Plant Methods 11, 1–11. 25774207 10.1186/s13007-015-0060-z PMC4359488

[CIT0035] Metzner R van Dusschoten D Bühler J Schurr U Jahnke S 2014 Belowground plant development measured with magnetic resonance imaging (MRI): exploiting the potential for non-invasive trait quantification using sugar beet as a proxy. Frontiers in Plant Science 5, 469. 25278947 10.3389/fpls.2014.00469 PMC4165221

[CIT0036] Miao Q Rahn JR Tourovskaia A Meyer MG Neumann T Nelson AC Seibel EJ 2009 Dual-modal three-dimensional imaging of single cells with isometric high resolution using an optical projection tomography microscope. Journal of Biomedical Optics 14, 064035. 20059273 10.1117/1.3275470

[CIT0037] Mooney SJ Pridmore TP Helliwell J Bennett MJ 2011 Developing X-ray Computed Tomography to non-invasively image 3-D root systems architecture in soil. Plant and Soil 352, 1–22.

[CIT0038] Ovečka M Vaškebová L Komis G Luptovčiak I Smertenko A Šamaj J 2015 Preparation of plants for developmental and cellular imaging by light-sheet microscopy. Nature Protocols 10, 1234–1247. 26203821 10.1038/nprot.2015.081

[CIT0039] Pajor R Fleming A Osborne CP Rolfe SA Sturrock CJ Mooney SJ 2013 Seeing space: visualization and quantification of plant leaf structure using X-ray micro-computed tomography. Journal of Experimental Botany 64, 385–390. 23307914 10.1093/jxb/ers392

[CIT0040] Palmer WM Martin AP Flynn JR Reed SL White RG Furbank RT Grof CP 2015 PEA-CLARITY: 3D molecular imaging of whole plant organs. Scientific Reports 5, 13492. 26328508 10.1038/srep13492 PMC4556961

[CIT0041] Pardo-Martin C Allalou A Medina J Eimon PM Wählby C Fatih Yanik M 2013 High-throughput hyperdimensional vertebrate phenotyping. Nature Communications 4, 1467. 10.1038/ncomms2475 PMC3573763 23403568

[CIT0042] Rieckher M Birk UJ Meyer H Ripoll J Tavernarakis N 2011 Microscopic optical projection tomography in vivo. PloS One 6, e18963. 21559481 10.1371/journal.pone.0018963 PMC3084718

[CIT0043] Sharpe J Ahlgren U Perry P Hill B Ross A Hecksher-Sørensen J Baldock R Davidson D 2002 Optical projection tomography as a tool for 3D microscopy and gene expression studies. Science (New York, N.Y.) 296, 541–545. 10.1126/science.1068206 11964482

[CIT0044] Staedler YM Masson D Schönenberger J 2013 Plant tissues in 3D via X-ray tomography: simple contrasting methods allow high resolution imaging. PloS One 8, e75295. 24086499 10.1371/journal.pone.0075295 PMC3785515

[CIT0045] Stuppy WH Maisano JA Colbert MW Rudall PJ Rowe TB 2003 Three-dimensional analysis of plant structure using high-resolution X-ray computed tomography. Trends in Plant Science 8, 2–6. 12523992 10.1016/s1360-1385(02)00004-3

[CIT0046] Tauriello G Meyer HM Smith RS Koumoutsakos P Roeder AH 2015 Variability and Constancy in Cellular Growth of Arabidopsis Sepals. Plant Physiology 169, 2342–2358. 26432876 10.1104/pp.15.00839 PMC4677887

[CIT0047] Telfer A Bollman KM Poethig RS 1997 Phase change and the regulation of trichome distribution in Arabidopsis thaliana. Development (Cambridge, England) 124, 645–654. 10.1242/dev.124.3.645 9043079

[CIT0048] Truernit E Bauby H Dubreucq B Grandjean O Runions J Barthélémy J Palauqui JC 2008 High-resolution whole-mount imaging of three-dimensional tissue organization and gene expression enables the study of Phloem development and structure in Arabidopsis. The Plant cell 20, 1494–1503. 18523061 10.1105/tpc.107.056069 PMC2483377

[CIT0049] van der Niet T Zollikofer CP León MS Johnson SD Linder HP 2010 Three-dimensional geometric morphometrics for studying floral shape variation. Trends in Plant Science 15, 423–426. 20541450 10.1016/j.tplants.2010.05.005

[CIT0050] Verboven P Herremans E Helfen L Ho QT Abera M Baumbach T Wevers M Nicolaï BM 2015 Synchrotron X-ray computed laminography of the three-dimensional anatomy of tomato leaves. The Plant Journal: for Cell and Molecular Biology 81, 169–182. 25319143 10.1111/tpj.12701

[CIT0051] Verveer PJ Swoger J Pampaloni F Greger K Marcello M Stelzer EH 2007 High-resolution three-dimensional imaging of large specimens with light sheet-based microscopy. Nature Methods 4, 311–313. 17339847 10.1038/nmeth1017

[CIT0052] Villani TS Koroch AR Simon JE 2013 An Improved Clearing and Mounting Solution to Replace Chloral Hydrate in Microscopic Applications. Applications in Plant Sciences 1, 1300016. 10.3732/apps.1300016 PMC4105042 25202549

[CIT0053] Volkov AG Pinnock MR Lowe DC Gay MS Markin VS 2011 Complete hunting cycle of Dionaea muscipula: consecutive steps and their electrical properties. Journal of Plant Physiology 168, 109–120. 20667624 10.1016/j.jplph.2010.06.007

[CIT0054] Walls JR Sled JG Sharpe J Henkelman RM 2005 Correction of artefacts in optical projection tomography. Physics in Medicine and Biology 50, 4645–4665. 16177495 10.1088/0031-9155/50/19/015

[CIT0054a] Wang C-N Hsu H-C Wang C-C Lee T-K Kuo Y-F 2015 Quantifying floral shape variation in 3D using microcomputed tomography: a case study of a hybrid line between actinomorphic and zygomorphic flowers. Front Plant Sci 6. 10.3389/fpls.2015.00724 PMC4564768 26442038

[CIT0056] Windt CW Gerkema E Van As H 2009 Most water in the tomato truss is imported through the xylem, not the phloem: a nuclear magnetic resonance flow imaging study. Plant Physiology 151, 830–842. 19710234 10.1104/pp.109.141044 PMC2754649

[CIT0057] Wong MD Dazai J Walls JR Gale NW Henkelman RM 2013 Design and implementation of a custom built optical projection tomography system. PloS One 8, e73491. 24023880 10.1371/journal.pone.0073491 PMC3762719

[CIT0058] Wuyts N Palauqui JC Conejero G Verdeil JL Granier C Massonnet C 2010 High-contrast three-dimensional imaging of the Arabidopsis leaf enables the analysis of cell dimensions in the epidermis and mesophyll. Plant Methods 6, 17. 20598116 10.1186/1746-4811-6-17 PMC2909956

