# Supplementary material for: Macro optical projection tomography for large scale 3D imaging of plant structures and gene activity
Source: J Exp Bot. 2016 Dec 26;68(3):527–38. doi: 10.1093/jxb/erw452 (PMC5441912; doi:10.1093/jxb/erw452)
Supplement: Supplementary Data [file erw452_Supplementary_Data.zip › Supplementary_video_legends.pdf]

Macro optical projection tomography for large scale 3D imaging of plant structures and gene activity

Karen Lee, Grant Calder, Christopher Hindle, Jacob Newman, Simon Robinson, Jerome Avondo and Enrico Coen

Supplementary video legends

Accompanying Fig. 4. Visualising mature flowers, leaves and fruits up to 60 mm tall and 45 mm deep in 3D with M-OPT

Supplementary Video S1
Pea (*P. sativum)* pod volume clipped to reveal peas inside. Transmission (red) and two emission channels, using a TXR filter (green) and a GFP1 filter (blue). Note air bubbles present between peas (red). Scan resolution of 62.5 µm/voxel.

Supplementary Video S2
Passion flower (*P. caerulea*). Fluorescent emission M-OPT of tissue autofloresecence captured via a GFP1 filter. Scan resolution of 62.5 µm/voxel.

Supplementary Video S3
Daffodil *(N. peoticus)* flower with transmission (red) and two emission M-OPT channels, using a GFP1 filter (green) and TXR filter (blue). Scan resolution of 40 µm/voxel.

Supplementary Video S4
Venus fly tap (*D. muscipula)*. Volume view of an immature trap showing the arrangement of veins in the valve and interlocking cilia at valve margins. Transmission (red) and GFP1 emission (green) channels. Scan resolution of 38.2 µm/voxel.

Accompanying Fig. 5. Comparison of OPT systems

Supplementary Video S5
Developmental series of snapdragon (*A. majus*) buds and flower. Buds of 0.5 mm to 1.2 mm in size were subject to fluorescent emission scans using a GFP1 filter collected with the MRC OPT scanner. Bud 1 from Fig. 5a, with a scan resolution of 3 µm/voxel. Bud 2 from Fig. 5b, with a scan resolution of 4.8 µm/voxel. Buds 2.5 mm to 7 mm deep and 2.6

mm and 10 mm tall were transmission OPT scanned with the Bioptonics 3001 OPT scanner. Bud 3 from Fig. 5c, with a scan resolution of 8.2 µm/voxel. Bud 4 from Fig. 5d, with a scan resolution of 24.4 µm/voxel. A mature flower 15 mm deep and 40 mm tall (Fig. 5e-f) was emission M-OPT scanned using a GFP1 filter with a scan resolution of 50.4 µm/voxel.

Accompanying Fig. 6. 3D biotic relationships between plants and insects

Supplementary Video S6

Venus fly trap (*D. muscipula)* and earwig (*F. auricularia)* visualised with M-OPT, with a scan resolution of 40 µm/voxel. Three M-OPT channels are combined. Multiple channels emphasise different features of the insect and leaf. Insect exoskeleton and internal regions of the head and thorax are visible in the transmission M-OPT channel (red), veins in leaf tissue, limbs and segmented regions of the earwig in green show tissue autofluorescence collected via the TXR filter. Another autofluorescence channel collected via the GFP1 filter is shown in blue in the mid-rib region of the trap (Fig. 6a-c). Zooming into the earwig inside the trap shows 3D structural details of the insect (Fig. 6d). In this case a single tissue autofluorescence M-OPT channel is viewed via a TXR filter (red), with a scan resolution of 6.5 µm/voxel.

Supplementary Video S7

Thrip (*Thysanoptera)* populations inside a snapdragon (*A. majus*) flower visualised with M-OPT (Fig. 6e-f). Two M-OPT channels are combined. Anthers, veins and sepals (yellow) imaged using emission M-OPT of tissue autofluorescence via a GFP1 filter. Petals (purple) and thrips (turquoise) imaged using transmission M-OPT. Scan resolution of 40 µm/voxel. Zooming in a thrip is visualised close to the floral veins and surrounded by trichome hairs (Fig. 6g), with a scan resolution of 6.5 µm/voxel.

Supplementary Video S8

Snapdragon flower *(A. majus)* and bee *(Apis)* visualised with M-OPT. Clipped M-OPT volume view through the centre of a bee inside a flower. Three M-OPT channels are combined. The transmission M-OPT channel (orange) highlights petals and the exoskeleton and internal organs of the bee. Tissue autofluorescence collected via the GFP1 filter is shown in green and was brightest in the insect's eyes and wings. Another autofluorescence channel collected via the TXR filter is shown in blue and is most prevalent in the exoskeleton. Scan resolution of 40 µm/voxel.

Accompanying Fig. 7. 3D gene activity in whole fixed and living Arabidopsis plants

Supplementary Video S9

AthB8::GUS gene expression in veins and roots of a fixed whole Arabidopsis plant (Fig. 7d), with a virtually dissected leaf showing leaf curvature (Fig. 7b, c). Scan resolution of 24.9 µm/voxel. Zooming into the stem scanned at higher magnification shows vein twisting (Fig. 7e). Scan resolution of 6.47 µm/voxel.

Supplementary Video S10

Living transgenic Arabidopsis plant with glabra 2::GFP fluorescence visualised with M-OPT (Fig. 7f, g). Multi-channel volume rendering with transmission M-OPT channel for plant structure (red) superimposed on the emission M-OPT channel (green). Scan resolution of 29.8 µm/voxel.

Supplementary Video S11

Visualising gene activity in 3D in a whole growing Arabidopsis plant. A transgenic Arabidopsis plant 27 days after sowing, expressing GFP clonal sectors after a 3.5 minute heat shock induction, imaged 7 days after treatment with emission M-OPT images collected via the GFP3 filter. After imaging the plant was returned to normal growth conditions, before imaging again the following day (Fig. 7i). This was repeated again 24 hours later (Fig. 7j). Scan resolutions of 19.7 µm/voxel (Fig. 7h), 24.9 µm/voxel (Fig. 7i) and 30 µm/voxel (Fig. 7j).
